# The Creeping Attachment Induced Technique (CAIT) in Natural and Restored Teeth: Case Reports with 24 Months of Follow-Up

**DOI:** 10.1155/2019/5828423

**Published:** 2019-01-29

**Authors:** Michele Perelli, Roberto Abundo, Giuseppe Corrente, Paolo Giacomo Arduino

**Affiliations:** ^1^Private Practice, Turin, Italy; ^2^Department of Periodontology, University of Pennsylvania, PA, USA; ^3^Department of Surgical Sciences, CIR-Dental School, University of Turin, Turin, Italy

## Abstract

This article describes a nonsurgical approach for treating gingival recessions and increasing gingival thickness around the natural teeth. Two female patients, presenting gingival recessions at the maxillary frontal teeth, were treated. Patient #1 had a discrepancy among the central maxillary incisors' gingival margin, and tooth UL1 needed to be restored. Patient #2 presented a buccal gingival recession at tooth UL3. In both cases, the sulcular gingival margin was gently disephitelized with a diamond bur leaving the soft tissue healing by itself. After 4 weeks, the procedure was reperformed. After 6 months, the gingival margins appeared thicker and a creeping attachment was achieved in both cases, obtaining gingival symmetry, related to the adjacent or contralateral teeth, and root coverage. Gingival asymmetry, gingival recessions, and gingival thickness may be improved by means of a guided gentle nonsurgical stimulation, providing creeping attachment in the natural and also restored teeth, with a healthy and stable tissue after 24 months of follow-up.

## 1. Introduction

In a healthy periodontium, the free gingival margin is normally located 1-2 mm coronally to the cementum-enamel junction (CEJ) and follows her convex contour around the tooth [1]. When gingival recession occurs, the free gingival margin is positioned apically, with the root's surface exposed to the oral environment. Every single patient has a personal gingival biotype which determines the thickness of the keratinized tissue. According to Rasperini's report, gingival thickness can be easily recorded by means of dedicated probes [2]. A well-known classification of gingival recessions is based on their coronoapical extension (with or without involvement of the mucogingival junction) and on the interproximal periodontal support [3]. Gingival recessions may have different causes, often associated with cervical defects of the dental enamel, radicular dentin, or both, due to caries, mechanical abrasions, chemical erosions, or abfractions [4]. If possible, root coverage could be obtained with a mucoperiosteal coronally or lateral-coronally advanced flap [5]. Histological data provides that healing of this modality of treatment may lead to a new connective attachment in the inner part of the flap and a long junctional epithelium in the coronal part [6]. Clinical study also reported the decreasing of gingival recession (REC) and physiological probing pocket depth (PD) in treated teeth [7]. The authors also describe that the adjunct of a connective tissue graft may thicken the gingival biotype and improve the performance in root coverage [8]. Recently, some authors have described the BOP (Biologically Oriented Preparation) technique, developed to shape the gingival tissue around a prepared tooth with a nonsurgical approach, which enables, by means of an induced coagulum and a provisional crown adapted to protect it under and along the gingival margin, to obtain gingival regrowth with a stability of its position over time [9].

The aim of the present manuscript is to describe the healing capacity of the periodontal tissue, when properly stimulated, in the presence of a gingival recession with a modified nonsurgical technique. REC, biotype, and free gingival margin stability were also recorded in this 2-year follow-up prospective clinical study.

## 2. Case Presentation

**Figure 1:**
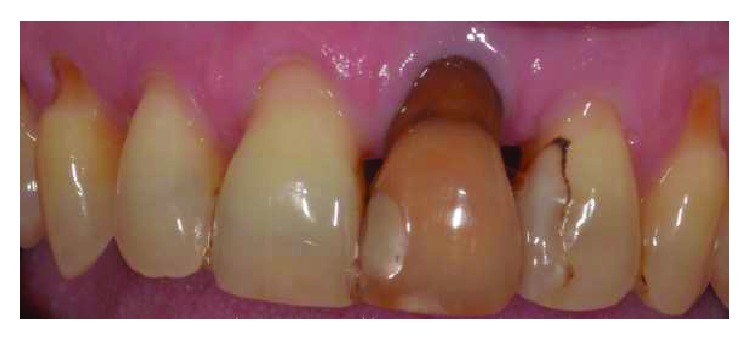
Patient #1: tooth UL1 presents an anaesthetic discoloration, cervical abrasion, thin gingival biotype, and free gingival margin discrepancy compared to the contralateral tooth.

Two female patients, not smokers, presenting gingival recessions at the maxillary teeth, were recruited and treated in the same private practice in Turin, Italy. Treatments were both performed in 2016 by the same clinician (M.P.). Patients did not report any contraindication to dental treatment. Patient #1 presented various maxillary gingival recessions; in particular, the left central incisor's free gingival margin was strongly misaligned compared to the contralateral tooth's gingival margin. Moreover, UL1 has been previously endodontically treated with an esthetical defect due to discoloration and radicular abrasion, having a buccal gingival margin almost 3 mm more apical than the contralateral (REC 3 mm), a 3 mm PD and showing a medium biotype (Figure 1). The treatment plane is aimed at reaching a pink and white aesthetic success by aligning the gingival parables of the central incisors and restoring the tooth with a ceramic crown.

**Figure 2:**
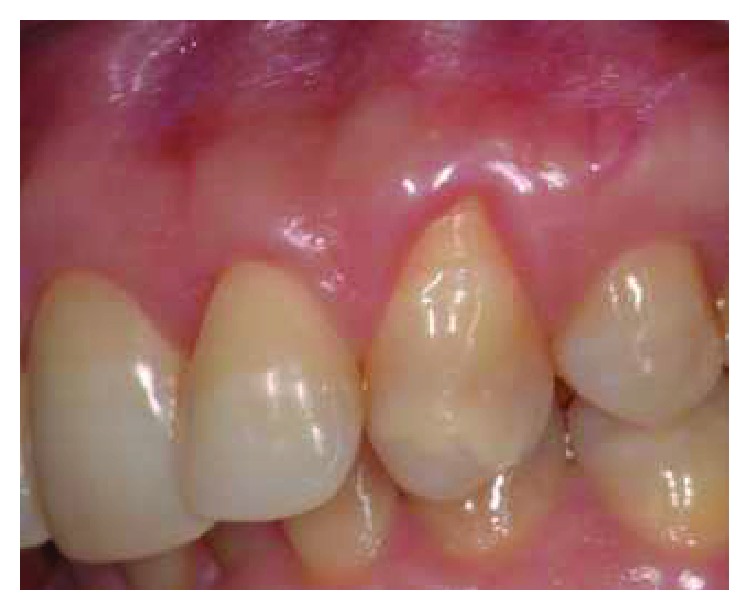
Patient #2: a buccal gingival recession of more than 2 mm is noticed in correspondence with UL3.

Patient #2 presented a single I Miller class recession in correspondence with the maxillary left canine. No radicular abrasion or abfraction were noticed, while a small coronal abrasion in correspondence with the cementum-enamel junction (CEJ) was noticed. REC was 4 mm, PD was 2 mm, and a gingival biotype was detailed as a medium (Figure 2). The treatment plane is aimed at a complete root coverage.

Both subjects initially received nonsurgical periodontal therapy, including oral hygiene instructions and supra- and subgingival scaling as required. Oral hygiene instructions were given by experienced dental hygienists. During each visit, subjects were instructed about oral hygiene maintenance at home. Such instructions were reinforced at each visit and were personalised when necessary. Instructions included modified bass technique with soft brushes (for 1 month) and a subsequent switch to medium brushes associated with interdental brushes. The patients were advised to change brushes every month and to change interdental brushes every 2 weeks.

### 2.1. Patient #1

Tooth UL1 had endodontic retreatment; when tooth reconstruction and delivery of the provisional crown were performed, the full-mouth plaque score (FMPS) and the full-mouth bleeding score (FMBS) indexes were both less than 25%.

**Figure 3:**
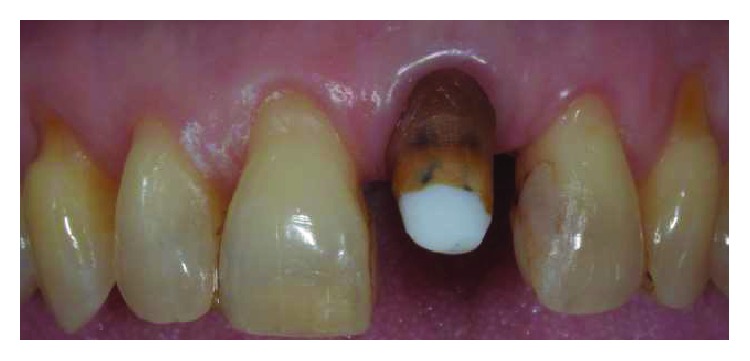
Central incisor was endodontically treated, restored with a composite post, and prosthetically prepared with a vertical finishing line.

**Figure 4:**
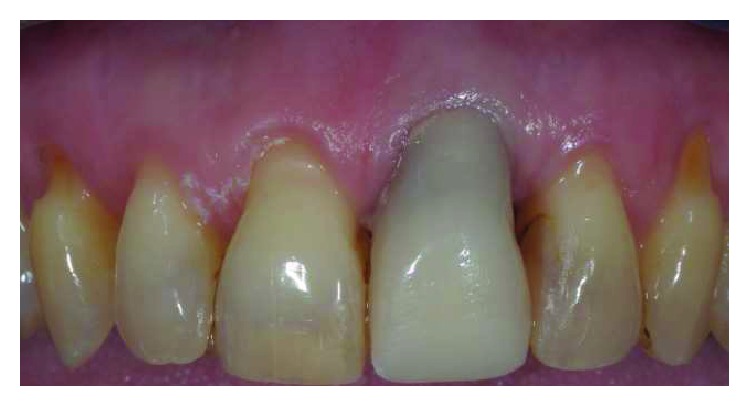
Temporary crown was delivered, and its cervical margin was located at the free gingival baseline position.

**Figure 5:**
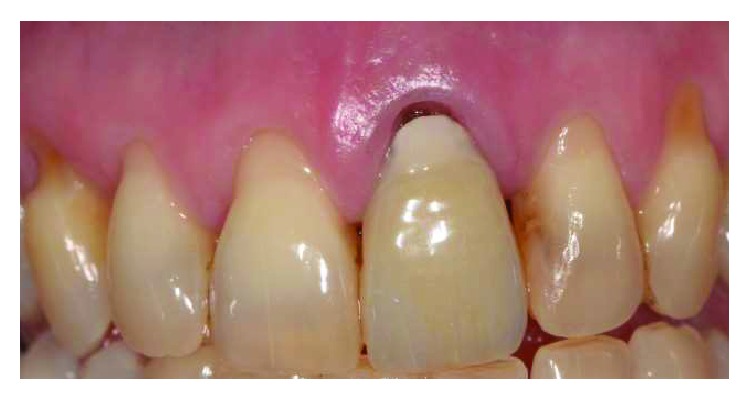
Temporary crown margin was 2 mm shortened.

**Figure 6:**
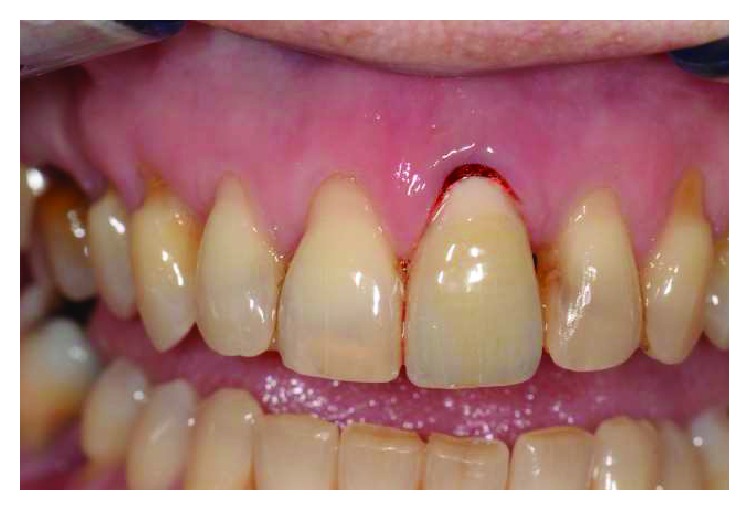
With diamond bur sulcus, junctional epithelium was gently removed, thus creating an induced inflammation.

**Figure 7:**
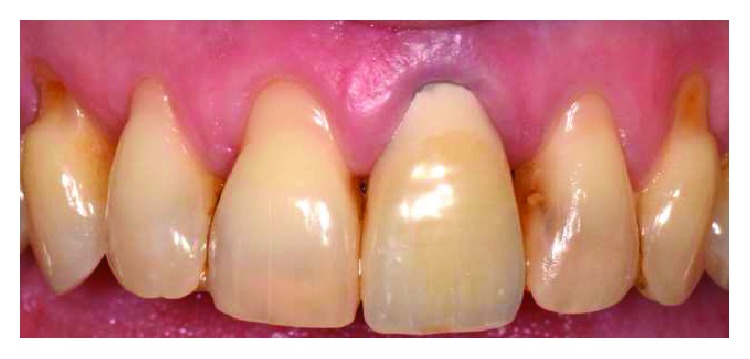
After 6 weeks, free gingival margin appeared thicker and an initial creeping can be noticed.

**Figure 8:**
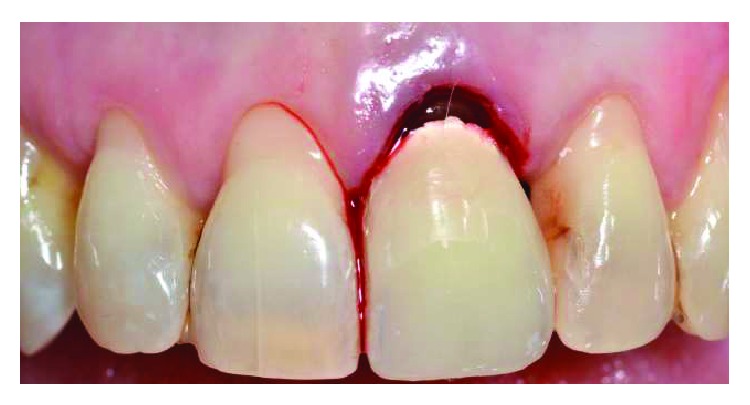
Temporary crown cervical margin was reshaped, mirroring the contralateral, and “stimulation” was performed again.

The tooth had vertical preparation, and temporary crown margins were adapted at first at the free gingival margin position (Figures 3 and 4). One week later after local anaesthesia, bone sounding was performed. The temporary crown buccal margin was 2 mm shortened, and the gingival epithelial components (sulcus and the upper part of the junctional epithelium) were gently disephitelized with a diamond flame bur (120-micron granulometry) and the root surface exposed to the oral environment planed and smoothed with manual curettes and washed with saline solution (Figures 5 and 6). The patient was instructed to clean the area with a 0.2% chlorhexidine spray solution twice a day for 4 weeks and not brushing the area. After 4 weeks, the gingival tissue appeared thicker, not inflamed, and an initial creeping was noticed. At this time, the patient started brushing with a soft toothbrush using the recommended technique. After 6 months, the gingival margin reached the temporary crown (Figure 7). Her buccal margin was shortened and aligned to the contralateral CEJ level, and the gingival margin was stimulated as before with the same postop chemical plaque control (Figure 8).

**Figure 9:**
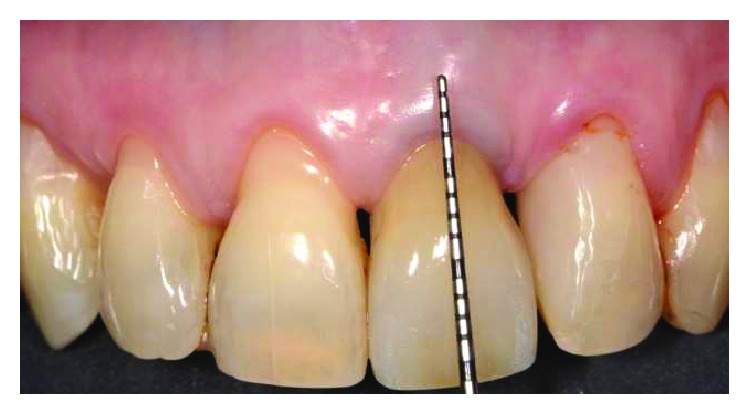
10 month picture: central incisors free gingival margin was at the same level; creeping occurred on UL1, with the thickening of the biotype.

**Figure 10:**
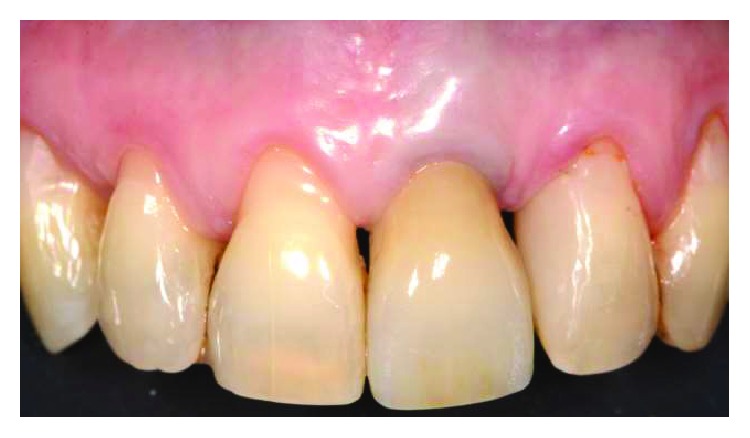
A definitive zirconia-ceramic crown was cemented (note the scalloped gingival architecture without scars or color mismatching). Control at 24 months of follow-up.

After 10 months, the maxillary central incisors presented free gingival margin at the same level with a different biotype, thicker on the treated gingiva. Impressions were taken and a zirconia-ceramic definitive crown was cemented to the tooth (Figure 9). At 2-year control, the free gingival margin's position was stable and the gingiva was in good health (Figure 10).

### 2.2. Patient #2

**Figure 11:**
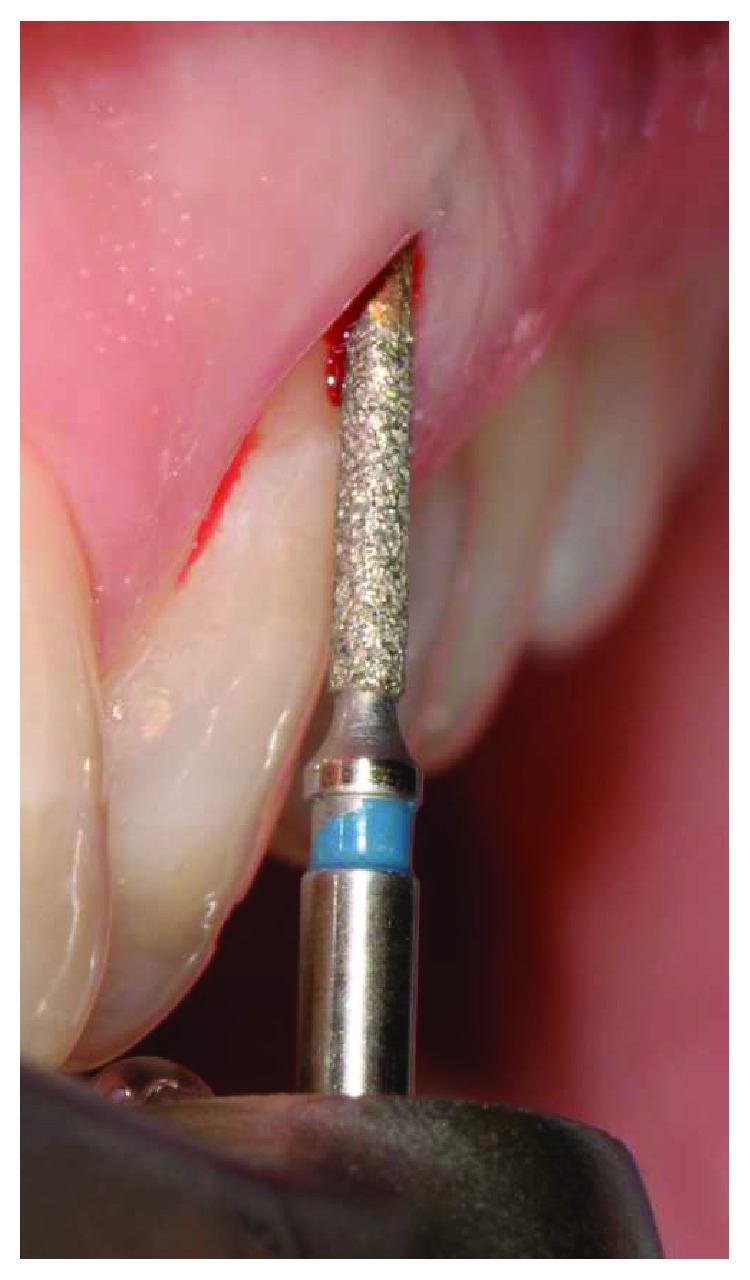
Radicular exposed surface was manually cleaned, and gingival epithelial components adjacent to the tooth were gently removed with a diamond bur.

**Figure 12:**
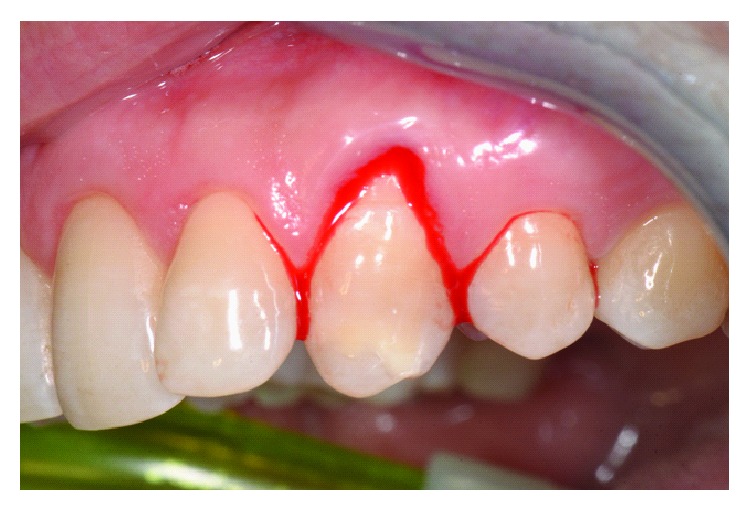
Marginal bleeding occurred with blood clot formation and stabilization.

**Figure 13:**
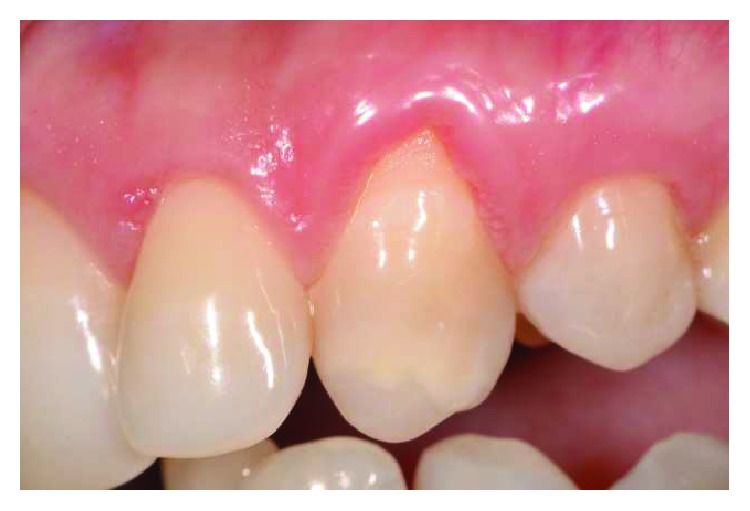
Gingival margin was thicker and again stimulated with the same protocol (4 weeks of follow-up).

**Figure 14:**
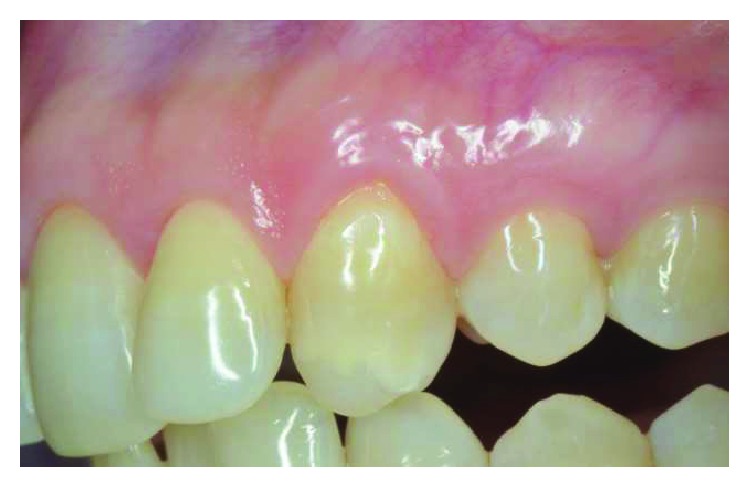
Complete root coverage was achieved by means of an induced creeping attachment at 6 months of follow-up; no scars and no tissue blending could be appreciated.

After professional scaling and root planning, FMBS and FMPS indexes were both less than 25%. After local anaesthesia, bone sounding was done. Using the same previous reported protocol, dental sulcus and coronal portion of the junctional epithelium were disephitelized with a diamond flame bur (120-micron granulometry), and the radicular surface was planed and smoothed with a manual curette (Figures 11 and 12). The patient was instructed not to brush the tooth for 4 weeks, using a 0.2% chlorhexidine spray twice a day for 4 weeks. Cold beverage and cold food were discouraged, in order to eventually prevent hypersensitivity. After 4 weeks, the free gingival margin appeared thicker with a slight creeping starting. In this second appointment, after local anaesthesia, the gingival margin was again stimulated as described before (Figure 13). The patient was instructed to start brushing the teeth after 4 weeks from this appointment. After 6 months, root coverage was reached, with a thick gingival margin and no pathological probing or inflammation (Figure 14). No dentin hypersensitivity was reported by the patient during this period.

**Figure 15:**
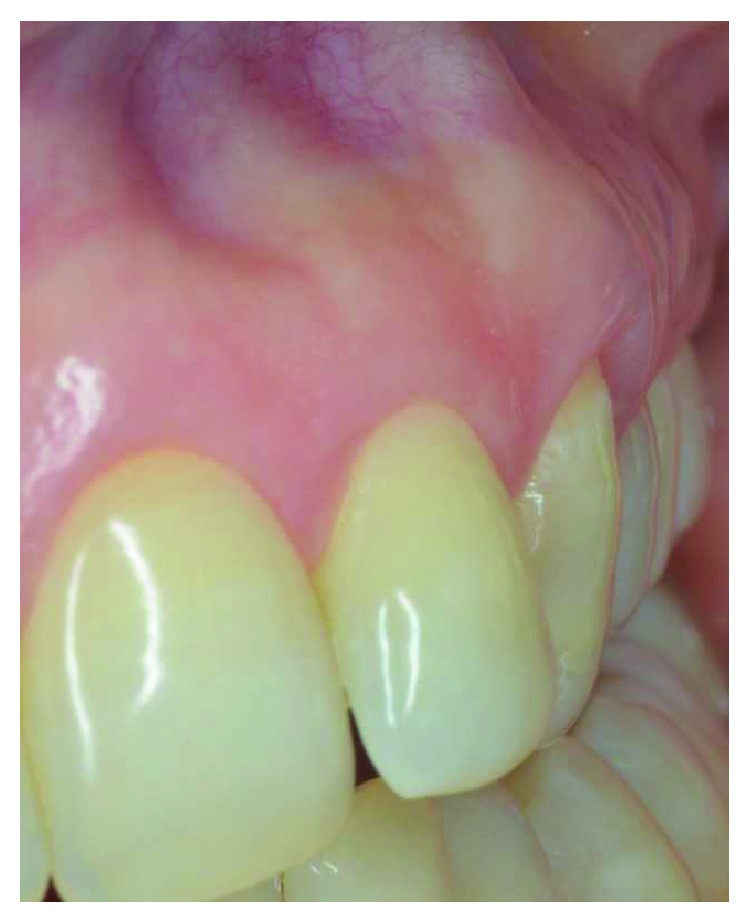
Stability of the root coverage on UL3 after 24 months.

In both patients, this technique demonstrated to be effective in gaining keratinized tissue and thickening the gingival biotype. In patient #1, the treated gingival margin had a coronal growth at the end of the treatment and the soft tissue marginal discrepancy among the incisors solved, with a creeping of almost 3 mm. There were no scars or tissues' blending; coverage of the exposed brown root and thickening of the gingiva were obtained. In patient #2, complete root coverage was achieved with a creeping of more than 2 mm also in this case. There was no dentin postop sensibility, and the PD was 2 mm. Results were stable at the 2-year follow-up (Figures 10 and 15).

## 3. Discussion

Gingival wound healing is a complex and dynamic process, usually involving different cellular types and metabolic mediators. In this process, epithelial and connective tissues interact and stimulated each other. The blood clot organization and stabilization is the first important phase described. Blood clot contains growth factors and defensive cells and is self-sustaining on the cleaned root surface. It is followed by the granulation phase [10]. During this step, the connective tissue, which is the sustaining component of the soft tissues, may thicken because the cells contained in the coagulum can promote its formation. To confirm this, the first observation in the study was the thickening of the gingival biotype, acting as a “connective” response to the technique. The last phase is tissue maturation which can hesitate to creeping attachment [11, 12], even with more than 2 mm growth in coronoapical dimension with the establishment of a physiological healthy probing depth. This is probably linked to a tissue rebound due to a new, healthy, and stronger connective tissue which ensures a firm contact between junctional epithelium and the root surface and “pushes” the gingival margin coronally.

In literature, the term “creeping attachment” has been described occurring during the second month after surgery [13] and continuing for 12 months and more, sometimes without a constant progression pattern, and recently has been reported also around implants [14, 15].

Most of the studies available in the literature involving the creeping attachment are related to the use of free gingival autografts [16, 17]. Matter mentioned that the factors that seem to have a definite influence on the phenomenon of creeping attachment around the teeth are the width of the recession, the position of the graft, the bone resorption, the position of the tooth, and the hygiene of the patient [12]. The physiologic mechanism behind creeping attachment on the natural teeth has not yet been fully elucidated, and it seems to be a multifactorial and unpredictable phenomenon. The proliferation of periosteum-derived connective tissue cells in response to surgical trauma, the characteristics of the donor tissue, its ability to bridge over the root surface and proliferate, and mature once transplanted seem to be crucial in determining whether the gingival margin will ultimately creep in a coronal direction [13].

It might be speculated that creeping attachment over the natural teeth might be more predominant due to the positive and favourable cellularity provided by the periosteum, and the capacity of the periodontal ligament to proliferate over a denuded root surface.

The reported stimulated nonsurgical biological approach may be useful to help clinicians in restoring dentogingival harmony and architecture both in the natural and restored teeth with good stability over time without any complications. An also important achieved result was the gingival thickening. In the present article, we have measured it with a dedicated probe and the results are in accordance with those reported by Agustin-Panadero and coworkers [18], describing a mean gingival thickening of 0.41 ± 0.28 mm for one-piece crowns and 0.38 ± 0.36 mm for FPD's, with gingival margin stability in all cases.

The findings of these two case reports must be investigated by studies with a larger number of patients, longer follow-up, and an evaluation in different gingival biotype and different clinical settings.
